# Applying photovoice and human-centered design to contextualize an adolescent micronutrient supplementation intervention in Mozambique

**DOI:** 10.1186/s41182-025-00768-8

**Published:** 2025-06-18

**Authors:** Sarah Bauler, Carmen Tse, Aicha Dos Santos, Lucilia Moises, Nicole Mbouemboue, Melissa Young, Joel Gittelsohn, Christine Marie George, Elli Leontsini

**Affiliations:** 1 World Vision International, London, UK; 2 World Vision Mozambique, Monapo, Mozambique; 3 Emory University Rollins School of Public Health, Atlanta, Georgia USA; 4 Johns Hopkins Bloomberg School of Public Health, Baltimore, Maryland USA

**Keywords:** Adolescent nutrition, Anemia, Human-centered design, Photovoice, Mozambique, Low- and middle-income countries, Formative research

## Abstract

**Background:**

Globally, iron-deficiency anemia is the most common micronutrient deficiency and a leading cause of disability-adjusted life years lost among adolescent girls 10–19 years of age. Adolescent girls’ voices are often excluded from shaping the interventions and policies designed to support them. We used participatory formative research methods—photovoice and adolescent-centered design (ACD)—to explore nutrition-related challenges, opportunities, and preferences among adolescent girls in Monapo District, Mozambique, and used the findings to contextualize a nutrition curriculum and supplement delivery platform.

**Methods:**

We purposively selected 16 girls from three rural and peri-rural secondary schools divided equally into two age groups (13–16 years and 17–20 years) and asked them to take photos of their food environment. Following a week of photo-taking, participants discussed their photos using the SHOWeD methodology in two workshops and in follow-up individual interviews. We also conducted three ACD group discussions with girls 13 to 20 years, each consisting of 10 to 12 participants, to explore consumption and supplement packaging preferences.

**Results:**

Thematic analysis of photos and transcripts showed that participants preferred locally grown foods and indigenous protein sources and were actively engaged in agriculture and household chores, highlighting opportunities for nutritional improvement. However, their nutrition was constrained by seasonal food shortages, inequitable household responsibilities compared to boys, and limited social capital. While school-based supplementation is the standard practice, participants strongly preferred to take supplements at home to avoid stigma and benefit from the comfort and privacy of their own homes. We used these insights to refine the adolescent nutrition curriculum and design a multiple micronutrient supplementation delivery platform.

**Conclusions:**

Photovoice provided rich visual data about the lived experiences of adolescent girls in a fragile and resource-constrained context, without the influence of an external researcher interpreting everyday realities, and elicited valuable insights into the barriers, opportunities, and potential improvements in nutrition programming. Integrating photovoice and ACD into program design can increase program acceptability and potential for effectiveness. This research also highlights the need to prioritize adolescent engagement and underscores the inadequacy of one-size-fits-all approaches, such as school-based supplementation programs.

## Background

Globally, iron-deficiency anemia (IDA) is the most common micronutrient deficiency and a leading cause of disability-adjusted life years (DALYs) lost among adolescent girls 10–19 years of age [1]. While anemia does affect boys, years lived with disability is higher among females than males between 10 and 84 years of age [2]. Heavy menstrual bleeding can exacerbate IDA and is often an underemphasized problem among adolescent girls [3]. Malnutrition, especially IDA, undermines girls’ cognitive ability and classroom performance, income potential, self-care abilities, and maternal and infant health outcomes. In Mozambique, 54.9% of girls between 15 and 19 years of age suffer from IDA [4].

Food insecurity, along with cultural practices and social norms, drives IDA among adolescent girls. Climate-related shocks—such as cyclones, floods, and droughts—along with political instability and violence, have driven food insecurity, contributing to over 2.7 million people being classified in Crisis (IPC Phase 3) or worse by the Integrated Food Security Phase Classification [5]. Mozambique also has one of the highest gender inequality indexes in the world [6], with more than half of girls dropping out of school before fifth grade [7]. Intra-household food allocation often favors men and boys [8]. Child marriage, early pregnancy, and high fertility rates further challenge adequate iron stores and the prevention of anemia. In Mozambique, 53% of girls marry before the age of 18, the fifth-highest percentage in the world [9]. Undernourished adolescent girls who marry and give birth early perpetuate the cycle of undernutrition, as micronutrient-deficient mothers are more likely to give birth to low birthweight babies [10].

School-based iron–folic acid supplementation (IFAS) programs have been implemented in low- and middle-income countries, including Mozambique, as a strategy to build iron stores before pregnancy and prevent IDA among menstruating adolescent girls and young women [11]. However, emerging evidence shows that multiple micronutrient supplementation (MMS) offers superior benefits compared to IFAS in reducing the risk for low birthweight babies (− 19%) and infant mortality at 6 months of age (− 29%) among pregnant women [12]. If Mozambique transitioned from weekly IFAS to daily MMS, an estimated 384,443 DALYs would be averted, making the intervention very cost-effective according to WHO guidelines [13]. Thus, a window of opportunity exists to develop contextualized delivery models of MMS and promote desired adolescent nutrition behaviors through education, to decrease IDA among adolescent girls and young women. While a national nutrition curriculum exists for primary school-aged children, no nationally endorsed curriculum is currently available for adolescents in secondary schools [14].

Globally, adolescents face barriers in decision-making regarding their nutrition needs. Their voices are often excluded from informing the design of health interventions and policies [15]. Addressing micronutrient deficiencies requires shifting the conversation from a biomedical issue to one of social justice and health equity, especially among vulnerable adolescents in low-resource settings [16]. Empowering adolescent girls to make informed decisions about their education, health, and nutrition is crucial, yet interventions aimed to improve health outcomes often exclude adolescents’ active participation in the design, implementation, and evaluation processes. However, there is a lack of formative research exploring how to effectively engage adolescents in these processes, limiting the development of nutrition interventions that truly reflect their needs and preferences. Recent studies have demonstrated that participatory video methods can empower adolescents—such as Ghanaian girls and Indigenous youth in India—by providing them with the opportunity to reflect and influence their nutrition behaviors and environment [17, 18]. The primary aim of this study was to identify the socio-ecological challenges and opportunities to consuming iron-rich foods among adolescent girls in Monapo District, Mozambique. A secondary aim was to assess the feasibility and value of using photovoice as a participatory research method in a fragile, resource-constrained, rural context. Findings from this formative research informed the refinement of an adolescent nutrition curriculum and the co-design of a multiple micronutrient supplementation (MMS) delivery platform.

## Methods

To explore nutrition-related challenges, opportunities, and preferences among adolescent girls, World Vision partnered with Mozambique’s Ministries of Health, Culture, and Education (MOH, MOC, and MOE) to implement two innovative formative research methods: photovoice and ACD group discussions. These activities were conducted between March 2023 and February 2024 in Monapo District, Nampula Province. The province faces significant challenges, with over 60% of its population living below the national poverty line—one of the highest rates in Mozambique [19]—and a stunting rate of 46.7% among children under five, the highest rate in the country [20]. In Monapo District, the majority of the population depends on small-holder farming, cultivating staple crops, such as maize and cassava [21]. Approximately 20% of the population in the district identifies as Muslim, with the majority identifying as Christian [22]. While the research findings were used to refine an adolescent nutrition curriculum and design an MMS delivery platform, this paper also seeks to provide insights into how youth-centered participatory approaches—particularly photovoice and adolescent-centered design (ACD)—can be adapted and implemented in a low-resource, fragile context.

### Photovoice

Photovoice is a participatory research method developed in the 1990s. Cameras are given to community members who typically do not have a voice in decision-making to allow them to document their experiences in a way that breaks down traditional communication barriers [23]. In this study, photovoice served as both as a formative research tool to explore barriers and opportunities related to consuming iron-rich foods, and as an advocacy tool to create opportunities for dialogue with influential individuals in the health and education systems. However, the focus of this paper is on how photovoice was used to elicit rich discussions and how its findings were leveraged to inform the refinement of an adolescent nutrition curriculum delivered to girls participating in school clubs.

The photovoice activity involved 16 girls from three rural and peri-rural secondary schools, divided equally into two age groups (13–16 years and 17–20 years) to capture varying perspectives by age. Each school provided a list of girls and their ages, from which we used a random number sheet to select participants based on predefined eligibility criteria, ensuring a diverse sample across socio-economic backgrounds. Participants were eligible if they were female, between 13 and 20 years of age, and enrolled in one of the three secondary schools identified for inclusion in the study. Each participant received a digital camera and a 1-h training session covering photography, ethics, and study objectives. The girls and their caregivers signed consent forms allowing the girls to participate. The girls also obtained photo release forms from their subjects to allow the photos to be shared publicly. Participants were instructed to take photos over a 1-week period, capturing their daily environment, meals, snacks, food sources, and purchasing locations, as well as images reflecting their aspirations and sources of happiness and sadness, to gain insights into the social and emotional environments influencing dietary behaviors and food access.

Following the week of photo-taking, two 5-h workshops were held (one per age group). Each participant selected her two or three most compelling photos (each girl took 50 to 60 photos), and discussed them using the SHOWeD methodology, which encourages reflection through a series of questions: (1) what do you *See* here? (2) What is really *Happening*? (3) How does this relate to *Our* lives? (4) *Why* does this problem, condition, or strength exist? (5) What can you do to *Educate* others about the problem, condition, or strength? (6) What can we *Do* about it? [24]. The workshops also explored participants’ understanding of healthy and unhealthy foods, their food preferences, and barriers and facilitators to accessing iron-rich foods. The workshops were recorded, and a notetaker documented the sessions in detail, later comparing their notes with the recordings to generate the transcripts.

Because some of the photos elicited strong emotional responses during the workshops, individual follow-up interviews were conducted with each participant to give them time and opportunity to explore the photos more deeply. During the individual interviews, each participant was asked to select one photo that best represented a challenge or opportunity related to health and nutrition.

The photovoice workshop and individual interview transcripts were translated from Portuguese to English and analyzed using MAXQDA 2022 Plus [25]. Our analysis was guided by the social–ecological model, a framework that highlights the interplay of factors influencing complex behaviors at multiple levels [26], including adolescents’ knowledge, attitudes, and emotions; household power dynamics; service accessibility; enforcement of child marriage laws; and education and health policies. A combination of deductive and inductive reasoning was used to develop a codebook encompassing descriptive, process, emotional, value, and concept codes. Horizontal analysis was employed to identify emerging themes across workshop transcripts, while vertical analysis focused on highlighting significant quotes and narratives. Coded segment reports were generated to systematically organize and categorize common themes and sub-themes. Insights were mapped to the adolescent nutrition curriculum objectives, guiding the adaptation of content and activities to ensure cultural relevance and practical application. Revisions were systematically tracked in an Excel spreadsheet, documenting original content, proposed revisions, and justifications for changes.

### Adolescent-centered design group discussions

Human-centered design—referred to in this study as ACD—is a participatory-based approach to problem-solving that involves users in the design phase by seeking to understand the needs, preferences, and behaviors of users to co-develop innovations to increase product uptake, acceptability, and impact [27]. We applied the ACD approach to co-design an MMS delivery model for adolescent girls enrolled in the intervention arm of a cluster randomized trial (CRT) [28]. One ACD group discussion was conducted at each of the three secondary schools in Monapo District, with 10 to 12 girls purposively selected for each session based on age (13–20 years) and school enrollment. Discussions explored preferred packaging for supplements, preferred location for supplement consumption (home versus school), and the best time to take the supplements (morning, afternoon, or evening). The ACD discussion guide was also informed by findings from the photovoice study.

Each discussion included a facilitator, a notetaker, and an observer. Sessions were recorded, and transcripts translated from Portuguese to English. Data were categorized into themes using Excel, documenting frequent challenges (“pain points”), actionable insights, and potential solutions to overcoming barriers identified by participants.

### Reflexivity

Reflexivity is integral to the research process, as it allows researchers to critically examine how their identities, assumptions, and experiences influence the research process [29]. To deepen this practice, our team engaged in structured, reflexive activities, posing questions about the historical, political, social, and religious contexts that shape our perspectives, and maintained detailed, analytical field notes. Following the ACD discussions, the research team conducted an activity in which each member created a personal outline detailing the traits and experiences that shape their identity. This prompted group dialogue about how our backgrounds might influence our interpretation of the data. The lead researcher also maintained analytic notes to reflect on her previous experiences in high-conflict contexts, and personal values may have shaped her interpretation of emerging themes.

### Ethical approval

Data collection activities were reviewed by the Mozambique Ministry of Health’s National Committee of Bioethics for Health (IRB00002657), which provided ethical oversight, and by the Institutional Review Board of the Johns Hopkins Bloomberg School of Public Health (FWA #00000287), which determined the activities to be public health practice.

## Results

### Indigenous food consumption and access to iron-rich foods

**Fig. 1:**
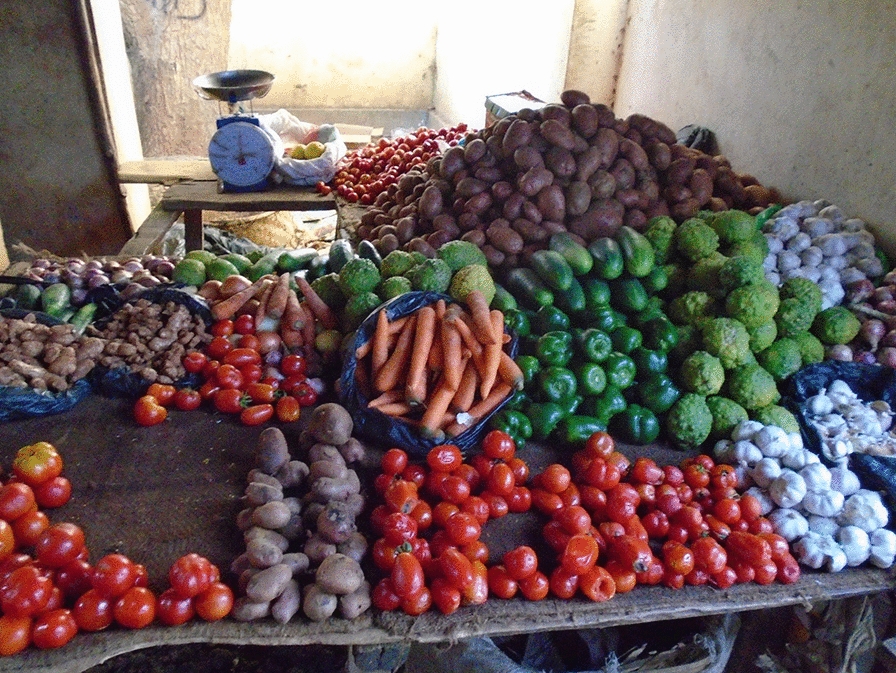
Photo from a 17-year-old girl showing the foods available in her community market

Findings from the photovoice activities, including workshops and individual follow-up interviews, revealed adolescents’ strong affinity for locally grown and wild-harvested foods (see Fig. 1). Commonly consumed indigenous foods included *chima* (cassava porridge), *maçanica* (fruit), rice, green beans, cassava, sugarcane, potatoes, banana, cucumber, peanuts, cabbage, okra, and sesame. Many of these frequently consumed foods are low in iron and other essential nutrients. Regarding protein and iron-rich animal foods, younger adolescent girls (13–16 years) frequently consumed field rats (roasted), while older girls (17–20 years) exhibited mixed acceptance. Chicken, fish, and goat, while accessible, were cost-prohibitive and thus consumed infrequently. Frogs (roasted), in particular, were strongly disliked and forbidden in Islam for consumption, while grasshoppers (roasted or eaten with tomato sauce) were more tolerated.

### Supporting mothers in agricultural activities

Adolescents expressed a strong desire to participate in agricultural activities, with most participants working on their family farms at least once a week. Mothers often managed their own plots of farmland, separate from those of their husbands, and adolescent girls supported their mothers in sowing and harvesting crops, such as cassava, tomatoes, rice, spinach, guava, and mangoes.

*“We usually go to the farm at 5 am and come back around 9 or 10 am on Saturdays.”* Participant 5

*“We usually plant tomatoes and manioc (cassava); my father has his field, but my mother’s field produces the most.”* Participant 1

### Cooking practices reflect socio-economic perceptions

**Fig. 2:**
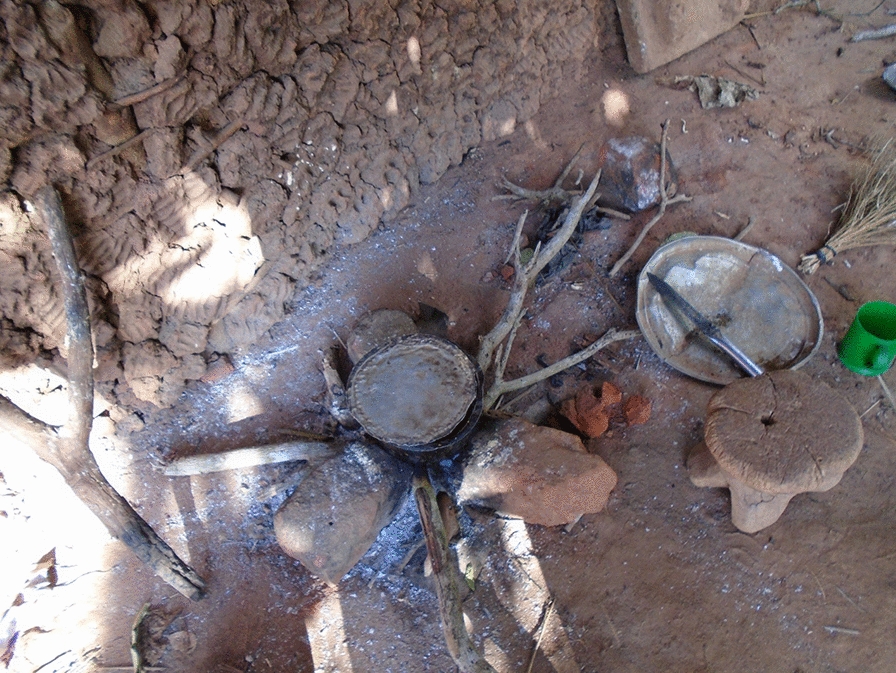
Photo from a 20-year-old girl showing how her family cooks on an open fire

Adolescent girls actively participated in preparing and cooking food, which often occurred outdoors over an open fire using charcoal or firewood. However, some participants conveyed feelings of shame through nonverbal cues when presenting their photos, particularly those depicting cooking over an open fire (see Fig. 2), which they associated with poverty. In contrast, images of certain household items, such as a television in one participant’s home, were associated with wealth. Participants deeply valued family, friendship, and community. Many girls captured and shared photographs of happy moments with loved ones, particularly during meals and outside their homes.

### Barriers to achieving aspirations

Participants expressed a strong desire to pursue education and careers to support their families, with teaching and healthcare being the most commonly mentioned professions. However, when asked to describe the obstacles to achieving their aspirations, such as becoming a teacher, a lack of high-status social capital was strongly associated with limited career opportunities. Interestingly, participants more often defined poverty not just as a lack of financial resources, but as a lack of connections to people of power and influence, which they felt was the most significant barrier to achieving their goals. Participants also identified early marriage as a pervasive problem in their communities and a reason why girls do not complete secondary school.

*“I have a dream of becoming a teacher (but) I’m afraid that I won’t be able to achieve my dream.”* Workshop 1, Participant 2.

Participants’ reflections on their aspirations and the structural barriers that constrain them revealed important contextual factors, such as poverty, early marriage, and limited social capital, that shape the broader social–ecological environment influencing adolescent girls’ dietary practices.

### Nutritional knowledge

Findings revealed that participants had limited knowledge of the nutrient content of certain foods. Some participants expressed a strong dislike of certain micronutrient-dense foods, such as carrots. Participants also demonstrated minimal awareness of the role of vitamin C as a nutrient that enhances iron absorption.

### Inequitable household responsibilities

**Fig. 3:**
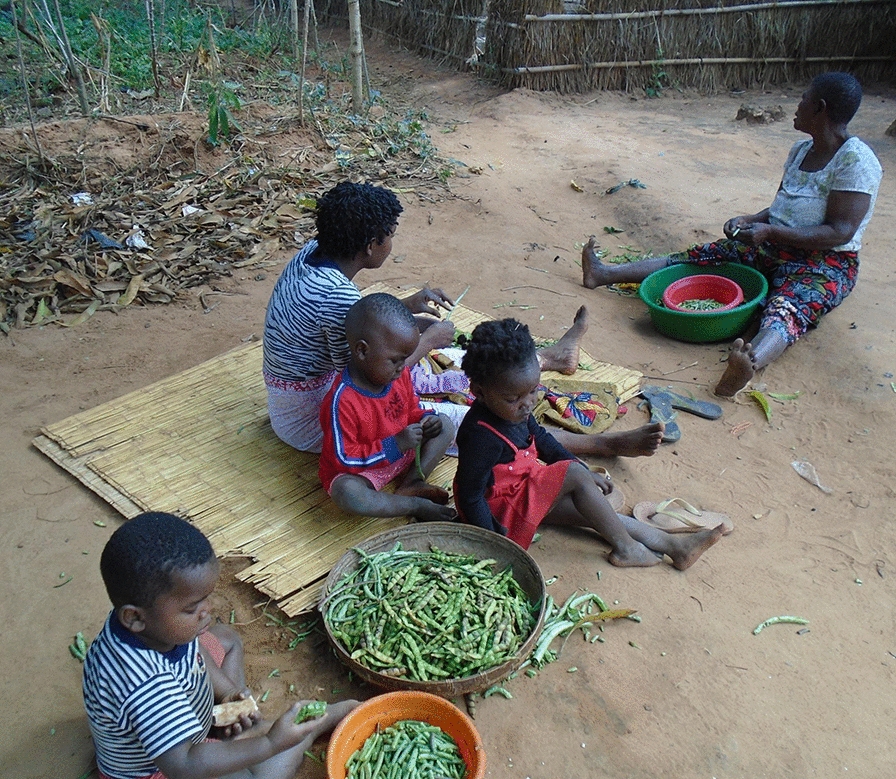
Photo from a 20-year-old girl showing how children, girls, and her grandmother shell peas

The concern that food was inequitably shared within the household, as highlighted in prior literature in sub-Saharan Africa [30, 31], did not emerge as a dominant theme in the qualitative analysis. However, disparities in household responsibilities between girls and boys were consistently found in the analysis. Girls were responsible for far more household chores than boys, including caring for younger siblings, planting and harvesting crops, collecting water, cleaning the compound, cooking, and washing clothes (see Fig. 3). Girls who attended school in the morning worked in the fields in the afternoon, while those who attended school in the afternoon worked in the fields in the morning. This division of schooling into morning and afternoon sessions is necessary to accommodate all students due to the limited number of teachers and schools. In comparison, boys were responsible for some agricultural activities, such as harvesting and planting, but were generally not responsible for cooking, cleaning, childcare, or collecting water and firewood.

*“… it makes me a little emotional, seeing this sister shelling peas to feed her family, together with her grandmother; it’s so difficult for a young woman like her to find herself doing this kind of thing…”* Individual interview

### Food insecurity

Several participants reported experiencing periods of food insecurity, especially during the lean months of March and April. Many adolescent girls shared that during these months, their daily meals consist of only cassava leaves. Participants identified obstacles to accessing sufficient food during this time of the year, including poor food market systems. Several participants took photos of children in their community who were visibly malnourished and shared that these photos made them the saddest, especially when parents neglected the health of their children, which they believed was exacerbated by food insecurity.

*“Producers sell all the products they grow without thinking about the times when resources become scarcer.” And “Sellers often increase the prices of products on the market [during this period].”* Participant 7

### Girls’ preferences for when supplements should be taken

Participants in the ACD group discussions overwhelmingly expressed a preference for taking iron supplements at home rather than at school. In Mozambique, the standard practice for iron supplementation involves distributing supplements to girls at school. However, all three discussion groups raised concerns about misconceptions regarding the purpose of the supplements. Specifically, participants reported that boys spread rumors that the girls were taking the supplements due to pregnancy. The girls felt that being allowed to take the supplements privately at home could help mitigate the embarrassment and stigma associated with taking them in a public setting. One participant recommended raising awareness among boys about the purpose of the supplements during the morning gatherings in the school courtyard, where students assemble to sing at the flagpole before starting classes. Another recurring theme was that taking supplements occasionally caused side effects, such as upset stomach and nausea. Participants noted that taking supplements at home would provide the advantage of allowing them to rest if they felt unwell.

*“There are those who take it (even) when it hurts (causes stomach pain), and there’s no bed to sleep in (at school), so it’s better to take it (supplement) at home so when it hurts, you can lie down for (a few) minutes, and it will pass.”* Participant 3

There was no consensus on the preferred time of day to take the supplements; all three discussion groups mentioned morning, afternoon, or evening, although consuming supplements at dinner was mentioned more frequently. Participants offered various suggestions for remembering to take supplements, including asking their mothers for reminders, taking them after bathing, and storing the supplements in their notebooks as a visual cue.

### High value for borehole water sources

**Fig. 4:**
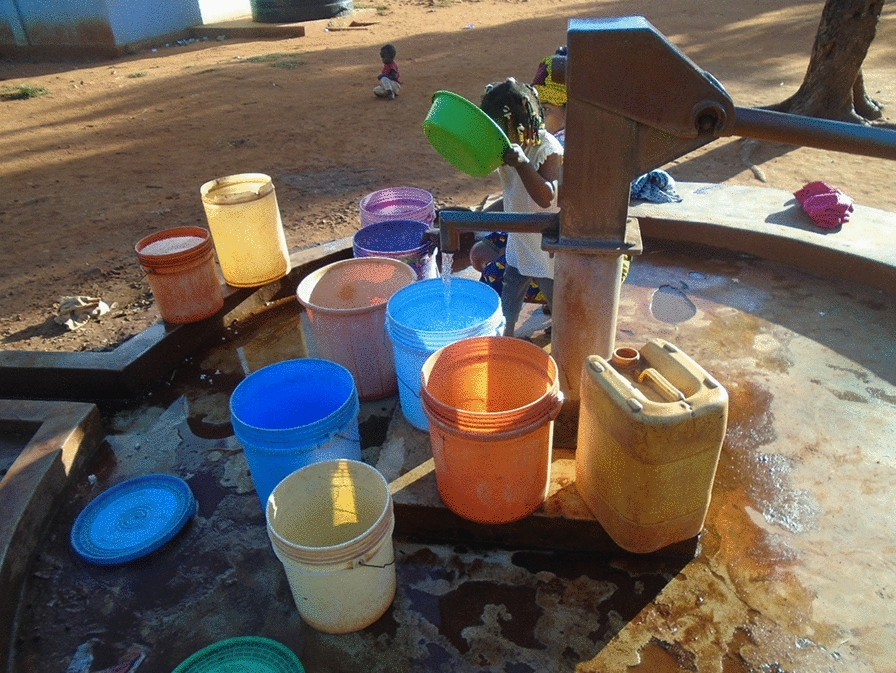
Photo from a 20-year-old girl showing her community well

Several participants took photos of borehole wells (see Fig. 4), which served as their primary sources of water. Water collection was primarily the responsibility of women and girls. Participants expressed that these wells were not only essential but also a source of happiness, as they provided reliable access to water in their communities.

### Girls’ preferences for supplement packaging

Regarding packaging, participants generally agreed that the MMS bottle label should feature distinct colors to differentiate it from family planning products. Specifically, they suggested green or black as preferable alternatives to the red commonly associated with family planning packaging. Some participants also expressed that the image of a woman on the MMS Vitamin Angels bottle did not resemble their perception of how a woman should be portrayed. Some participants also felt their parents should be informed about the purpose of the supplements and that relevant information be provided to them.

*“We don’t know the name of the pill we were given at school; our guardian should know what it is; what the pill does and the name.”* Participant 3

## Discussion

**Fig. 5:**
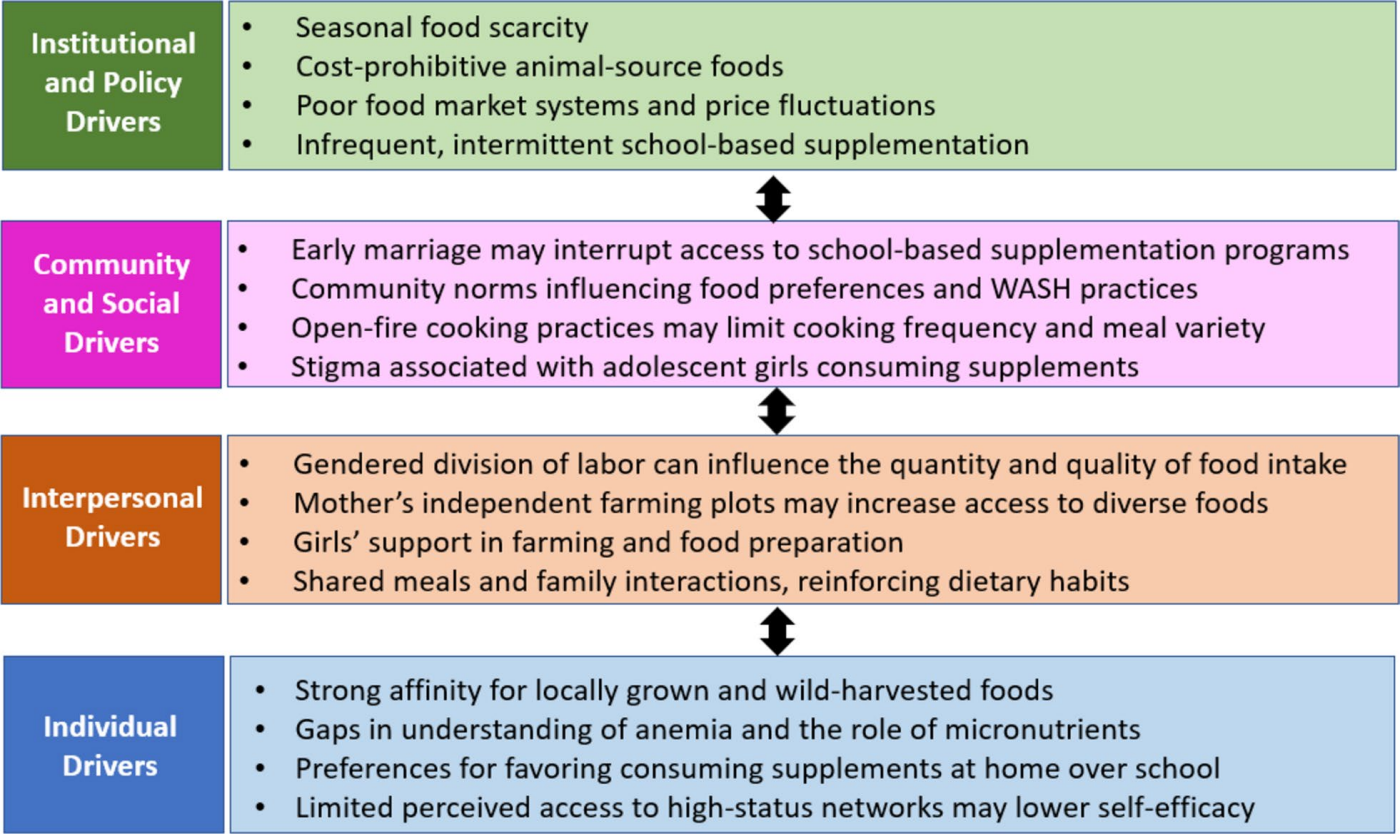
Conceptual framework illustrating the social and ecological drivers of dietary practices among adolescent girls in Monapo District, Mozambique

This research offers practitioners and policy maker’s clearer insights into the social and ecological factors influencing dietary practices among adolescent girls in Monapo District, as illustrated by the conceptual model in Fig. 5. Our conceptual framework is primarily grounded in the empirical findings that emerged from the photovoice and ACD activities; however, the social–ecological model has been widely applied in the literature to understand adolescent nutrition and gender norms [26]. The findings from our formative research align with broader trends in the adolescent nutrition literature from sub-Saharan Africa, highlighting that IDA is shaped not only by dietary intake but also by the social and emotional environments that influence girls’ nutrition-related behaviors. Gender norms and seasonal food insecurity, both illustrated in our framework, have also been reported as key barriers to accessing and consuming iron-rich foods among adolescent girls in Ethiopia, Ghana, and South Africa [30, 31].

We have used this conceptual framework to guide the refinement of the adolescent nutrition curriculum and to develop an MMS delivery platform. Changes to the curriculum, as illustrated in Table 1, included adding sections to clarify that IFAS and MMS are solely for preventing anemia and not for preventing pregnancy. A new section was added to address myths and rumors spread by boys about the supplements, including a brainstorming activity to help girls develop strategies to counter these misconceptions. The refined curriculum includes practical cooking demonstrations using affordable, locally sourced Indigenous and high-iron foods. These sessions aim to develop menus that can be used at home and at school. Participatory cooking demonstrations have been found to improve diet diversity, improve nutritional knowledge, and increase self-efficacy [32, 33]. In addition, the curriculum now emphasizes intergenerational and traditional knowledge sharing to reinforce connections between generations, which is often maintained through the handing down of recipes [34] and supporting mothers in farming activities. The value of consuming wild-harvested foods was added to the curriculum, as rodents and frogs contain substantial levels of bioavailable heme iron—comparable to farmed meats—and grasshoppers are rich in iron [35–37]. As cassava was found to be a commonly consumed and well-liked food, a section was added on the proper techniques for peeling, washing, soaking, and boiling cassava to reduce toxicity.

**Table 1:** Contextualization of the adolescent nutrition curriculum

Formative research findings	Refinement to curriculum
Adolescent girls are confused about the purpose of taking supplements and confuse them with birth control pills	Added a section that iron supplements do not have anything to do with preventing pregnancy and only to help prevent anemia
Adolescent boys spread rumors and tease girls who take supplements at school	Added a section in the curriculum on how to address rumors and how to sensitize boys on the purpose of the supplements
Adolescent girls have limited access to and consumption of iron-rich foods	Included more time for cooking demonstrations and recipe creation using locally available and/or Indigenous foods with high-iron content
Cassava was a commonly consumed food. However, adolescent girls can be exposed to toxins in cassava if not cooked properly	Included cooking demonstrations on how to cook cassava properly
In discussions of their future aspirations, some adolescent girls shared their dreams of becoming a schoolteacher	Included an example of achieving the dream of becoming a schoolteacher as a scenario for adolescent girls to reflect on how they would celebrate in that situation
Body image was not mentioned in the workshops	Healthy body image session was removed
Rats (roasted) are a common and preferred local food	Included rats in the examples within the sessions as a source of wild-harvested foods high in iron
Cooking food over fires was associated with poverty and shame, and may limit cooking frequency and meal variety	Cooking demonstrations are conducted over both cooking stoves and fires to ensure lessons are feasible and to increase social acceptability of girls who use fires for cooking
Girls support their mothers and grandmothers in farming and food preparation	Included a section on the importance of sharing their nutrition knowledge and recipes with their mothers and grandmothers
Shared meals and family interactions reinforce dietary habits, especially with mothers and grandmothers	Included a section on encouraging the girls to learn from their mothers’ and grandmothers’ local wisdom and food knowledge
Food insecurity, especially during the lean season	Added a section on the value of school, community, and home gardens
Cost-prohibitive animal–source foods	Added a section on the value of harvesting and consuming wild animals (rats and frogs) and grasshoppers
Inequitable division of chores between girls and boys	Highlighted in the curriculum how gender inequities influence health and nutrition and outcomes and a discussion on how these inequities can be tackled
Early child marriage	Highlighted in the curriculum is the link between early marriage and poor nutritional outcomes
Career aspirations hindered by a lack of social capital	Added modules on the service project to build confidence and increase social connections
Borehole wells highly valued by adolescent girls	Added a section on the importance of water treatment methods and safe water storage practices

The curriculum now highlights the role of school, community, and home gardens, as small gardens have been found to increase vegetable intake and improve behavioral determinants and food availability and diversity [38, 39]. Despite the high value placed on borehole wells in communities and schools, water quality testing showed that the water they provided was not consistently safe for drinking. Fecal coliforms and *E. coli* levels exceeded 100 UFC/100 ml in two of the three school borehole wells tested. Content was added to the curriculum on the importance of water treatment methods, such as filtering, chlorine tablets, and boiling, to remove harmful pathogens from water. Safe water storage practices are also highlighted, including the use of clean cups for drinking, as supplements are commonly taken with water in plastic cups or bottles.

Sessions were also added on gender inequities and early marriage to improve knowledge of how these social issues impact health and nutrition outcomes. Girls who marry early are more likely to drop out of school in Mozambique [40], disrupting their access to school-based micronutrient supplementation and nutrition programs. The curriculum explains how undernourished adolescent girls are more likely to experience early pregnancy, with insufficient time to rebuild iron stores, which increases the risk of giving birth to low birthweight and undernourished infants, thereby perpetuating the intergenerational cycle of anaemia. [10] To address myths and teasing perpetuated by boys related to consuming supplements, we added a session on how to demonstrate assertive and non-violent communication skills to improve conflict resolution skills. We also added a session on discussing gender differences, as gendered division of labor, where girls are responsible for more household chores than boys, can lead to time poverty and negatively influence the quantity and quality of food intake. [41].

To promote agency and social capital, we incorporated a service project module conducted in collaboration with school administration staff and community leaders to build adolescents’ skills and confidence in designing, implementing, and evaluating community-based nutrition improvement initiatives. At one school, the clubs collaborated to plant a school garden; at another, they constructed a nutrition classroom; and at the third school, they built latrines to improve access to sanitation. Providing social action opportunities for adolescents has been found to increase their resiliency and self-efficacy [42]. Body image was not mentioned in the workshops, and it did not appear to be an important driver for dietary and physical activity behaviors, thus this lesson plan was removed from the curriculum. Embedding service projects, community cooking demonstrations, and lessons on gender norms, alongside content on individual dietary practices, allows us to address determinants of IDA across the SEM. This approach to developing a participatory curriculum by integrating behavior change messaging with strengthening structural and social supports to improve equity and participant engagement has also been exemplified in programs such as one program implemented in Malawi and Tanzania [43].

ACD participants overwhelmingly preferred to take supplements at home rather than at school. This preference was further validated by the CRT baseline survey, which found that 64.3% of respondents preferred taking supplements at home, and 83.9% preferring to take them after dinner. These findings reinforce the challenge of the standard school-based supplementation model, which requires consuming supplements in the morning or early afternoon. At-home supplementation strategies also provide an opportunity to reach out-of-school girls, a crucial consideration in Mozambique given that more than 50% of girls leave school before they complete fifth grade [44]. Providing each girl with a 90-tablet supplement bottle at home (provided by the MOH or procured through local markets) could enable girls to maintain consistent supplement intake during weekends, extended school breaks, and periods of political turmoil when schools may be forced to close. At-home supplementation strategies should also assess the potential for supplement sharing among household members and ensure proper storage practices, including keeping bottles securely closed and out of reach of young children.

One advantage of school-based supplementation programs is that teachers and health officials can directly monitor the consumption of supplements by girls, thereby enhancing adherence and compliance. In contrast, at-home supplementation places the responsibility on the girls themselves to remember and adhere to the prescribed schedule—daily for MMS and weekly for IFAS—which may pose challenges without proper support and reminders [45]. Adolescence is a period of rapid neurocognitive development, often marked by impulsivity and poor response to failure and setbacks [46], making the use of reminder cues and other supportive strategies essential to ensure consistent adherence to supplement regimens. To address this issue, ACD participants brainstormed strategies to enhance adherence to at-home supplementation schedules, which will be evaluated in the CRT.

Our research highlights the value of meaningfully engaging adolescents in program design and explores whether one-size-fits-all approaches adequately meet their diverse needs. Policies should consider alternative delivery models that align with adolescents’ preferences, such as at-home supplementation, while addressing broader issues, such as food scarcity and inequitable gender norms. At-home supplementation strategies could also be an effective delivery platform to reach millions of girls in Mozambique who are not in school, including those affected by political insecurity. Integrating participatory methods into policy development could lead to more inclusive and effective solutions. Policymakers should recognize adolescent girls as key stakeholders and leverage their insights to inform strategies that promote nutrition, education, and empowerment, ultimately contributing to long-term improvements in public health outcomes. Finally, it is important to note that the nutrition curriculum and supplement delivery model is more targeted toward downstream determinants affecting IDA than upstream determinants—the political and institutional drivers of nutrition. This study was embedded into a larger gender empowerment project aimed at enforcing laws against child marriage and sexual and gender-based violence. Behavior change interventions (downstream) are more likely to be sustained when they are integrated with programs that seek to cultivate an enabling environment and address distal factors impacting health and nutrition outcomes [47].

Regarding the feasibility and value of the formative research methods, we learned many lessons. Despite receiving only a brief, 1-h training on basic photography techniques and having no prior experience with smartphones or cameras, participants demonstrated remarkable skill in capturing high-quality, clear images. Initial concerns from secondary school teachers regarding the potential for theft or damage of cameras were mitigated by the participants’ careful handling and storage of the equipment in protective cases. Notably, all cameras were returned intact and fully functional. During workshops, photos were displayed via projection, which facilitated group discussions; however, the availability of printed copies would have enhanced the process of image categorization and thematic analysis. We recommend incorporating printed photographs into photovoice workshops when resources permit, as this practice can improve participant engagement and streamline analytical procedures.

We found that sharing photos can sometimes evoke feelings of shame among participants, particularly when images depict poverty-related realities. Some participants conveyed feelings of embarrassment regarding their photos through nonverbal cues, especially images associated with poverty, such as cooking on an open fire. This finding underscores the potential sensitivity of seemingly neutral subjects, such as the food environment, and highlights the need for photovoice implementers to approach these discussions with care. To navigate such emotional responses effectively, workshop and group facilitators should receive specialized training in handling sensitive topics with empathy and cultural awareness. Individual interviews offer a complementary approach, providing participants with a more private setting to explore deeply personal or potentially stigmatizing issues. We recommend integrating both group discussions and individual interviews in photovoice projects to broaden the insights gained while ensuring participants’ comfort and dignity.

Finally, methodologically, our use of photovoice aligns with findings from similar studies in sub-Saharan Africa, where this participatory approach has proven effective in generating context-specific insights related to nutrition, gender, and adolescent health [48–50]. These photovoice studies in Ghana and Ethiopia allowed participants to share their lived experiences, providing youth-centered insights into food insecurity, nutrition preferences, and behaviors.

### Strengths and limitations

A key strength of this study is its use of simple tools, such as cameras, which allowed participants to act as data collectors, analysts, and consultants, reducing the need for extensive staff or infrastructure. The approach generated rich, actionable insights without requiring complex analysis or high costs. By directly engaging participants and integrating visual storytelling and design thinking, photovoice and human-centered design approaches can provide rich information with minimal resources, making these approaches particularly well-suited for resource-limited settings.

As with all qualitative and formative research studies, the primary limitation is that its findings are context-specific, derived from a small group of participants, and may lack representativeness to the broader population. Instead, our qualitative research should be evaluated for rigor based on established criteria; transferability, confirmability, and dependability [51]. Only the readers of this paper can assess the applicability and transferability of the findings to other contexts; while we strived to provide rich contextual details, space constraints in this paper have limited some descriptions. To ensure confirmability and dependability, we employed triangulation to cross-verify our findings; however, the limited body of scientific evidence on the determinants of IDA among adolescents in Mozambique constrains our validation. In addition, user-centered techniques, while valuable for tailoring solutions to individuals’ immediate needs, may overlook broader systemic factors, such as government policies or financial constraints that may hinder the adoption and scalability of the solution. Such limitations highlight the need to balance user-centered insights and preferences with consideration of structural, economic, and organizational realities. To address this, the second phase of the study—the CRT—will evaluate the acceptability and impact of the contextualized nutrition curriculum on improving diet diversity and anemia-related knowledge and compare IFAS versus MMS, measured through hemoglobin levels.

## Conclusions

The findings from this study demonstrate the importance of adolescent-centered, participatory formative research approaches in contextualizing and designing effective nutrition interventions. Adolescents, particularly girls, face unique barriers to nutritional health, including gender-based inequities, stigma, and limited social connections with people of power and influence who can create opportunities for gainful employment. Using participatory methods, such as photovoice and ACD, this study provided critical insights into adolescents’ lived experiences and preferences, which informed the development of a culturally relevant adolescent nutrition curriculum and an innovative MMS delivery platform. These approaches created opportunities for adolescents to share their lived experiences, concerns, and preferences, potentially fostering a sense of agency and empowerment. Integrating these methods into program design can increase their acceptability, potentially increasing uptake and sustainability in similar low-resource settings.

## Data Availability

No datasets were generated or analysed during the current study.

## Abbreviations

ACD: Adolescent-centered design
CRT: Cluster randomized trial
DALY: Disability adjusted life year
IDA: Iron deficiency anemia
IFAS: Iron–folic acid supplementation
MOC: Ministry of Culture
MOE: Ministry of Education
MOH: Ministry of Health
MMS: Multiple micronutrient supplementation
WHO: World Health Organization
